# First person – Nicola Rossi

**DOI:** 10.1242/bio.058721

**Published:** 2021-04-15

## Abstract

First Person is a series of interviews with the first authors of a selection of papers published in Biology Open, helping early-career researchers promote themselves alongside their papers. Nicola Rossi is first author on ‘Oviductal fluid counterbalances the negative effect of high temperature on sperm in an ectotherm model’, published in BiO. Nicola is a PhD student in the lab of Margarita Chiaraviglio and Gabriela Cardozo at Instituto IDEA, Córdoba, Argentina, studying the effects of global warming on seuxal selection and social mechanisms in a lizard species native to South America, *Tropidurus spinulosus*.


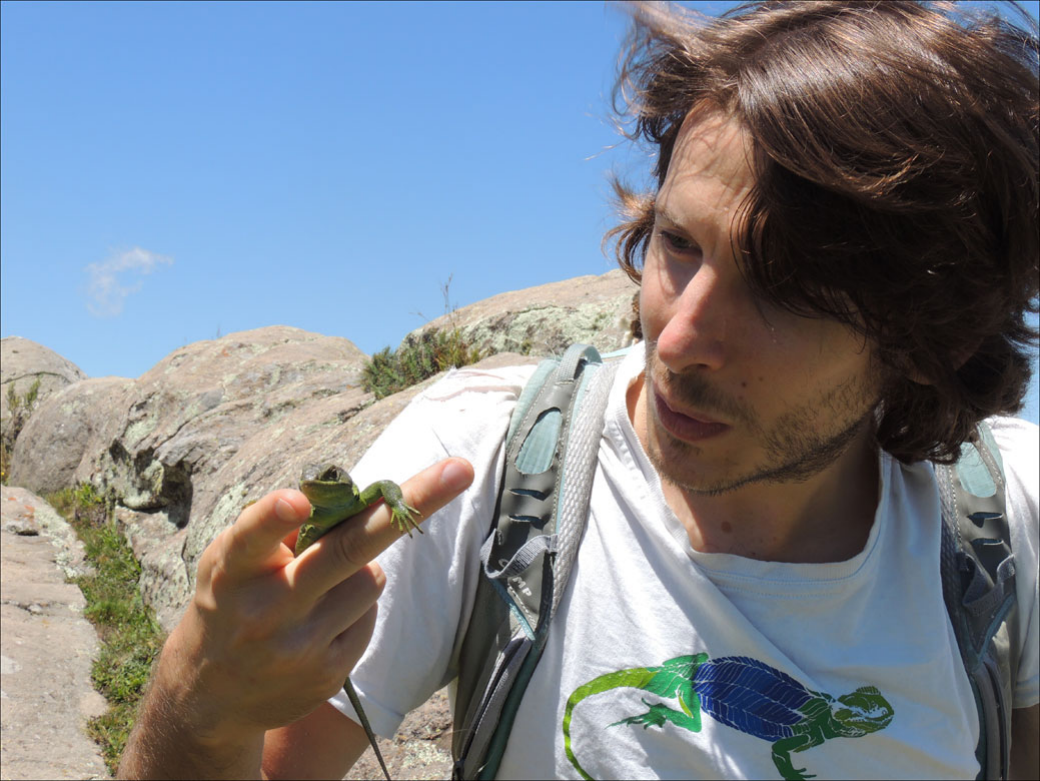


**Nicola Rossi**

**What is your scientific background and the general focus of your lab?**

I earned my Masters Degree at the University of Ferrara in Italy by defending a thesis on home range and habitat selection of the Common Adder (*Vipera berus*) in the Archipelago National Park (Turku, Finland) under the supervision of Dr. Pälvi Salo.

I am currently doing my PhD at the Behavioural Biology laboratory of the National University of Córdoba in Argentina under the guidance of Dr. Cardozo Gabriela and Dr. Chiaraviglio Margarita. Our laboratory aim is to elucidate proximal and ultimate causes of the diversity of patterns and processes that underlie the evolution of reproductive strategies, contributing to scientific knowledge by building hypothesis in the field of Evolutionary Ecology and Conservation Biology.

**How would you explain the main findings of your paper to non-scientific family and friends?**

Reproduction is one of the main events in a species’ life cycle. In several vertebrates, the reproductive dynamics are shaped by complex interplay between the sexes and environmental factors. Sperm cells are fundamental cogs in the reproductive machine, spermatozoa motility is of vital importance and the condition of the swimming environment may either hamper or enhance it. Temperature is a key factor in some species with internal fertilization as it can harm sperm cells, thereby reducing their motility. In light of global warming, this is relevant especially for cold-blooded animals due to their strict relationship with temperature. Our study evaluated whether the fluids of the female reproductive tract aid in preserving sperm motility when temperatures increase, in a South American native lizard species *Tropidurus spinulosus*. The results suggest that the action of the oviductal fluid will be fundamental in preserving sperm motility when environmental and body temperatures of females are high.

**What are the potential implications of these results for your field of research?**

Our study was the first to test the effect of high temperatures and the role of the oviductal fluid in maintaining sperm motility at high temperatures in Squamata. This paves the way to several research questions such as the mechanisms involved in the maintenance of motility, the possible role of the oviductal fluid in the cryptic female choice and how this may be modified by temperature.

**What has surprised you the most while conducting your research?**

Lizards and in particular our species are full of surprises. It never ceases to amaze me to see how social these beings are (many people still think the opposite) and how complex their reproductive dynamics can be.

**What, in your opinion, are some of the greatest achievements in your field and how has this influenced your research?**

The achievements that are marking our laboratory current topics are related to the ongoing discovery of fine-scale reproductive mechanisms, such as cryptic female choice and sperm competition mechanisms. I think that delving deeper into these issues will give us the necessary insights to understand current animal reproduction and make projections in relation to future issues such as climate change.

**Figure:**
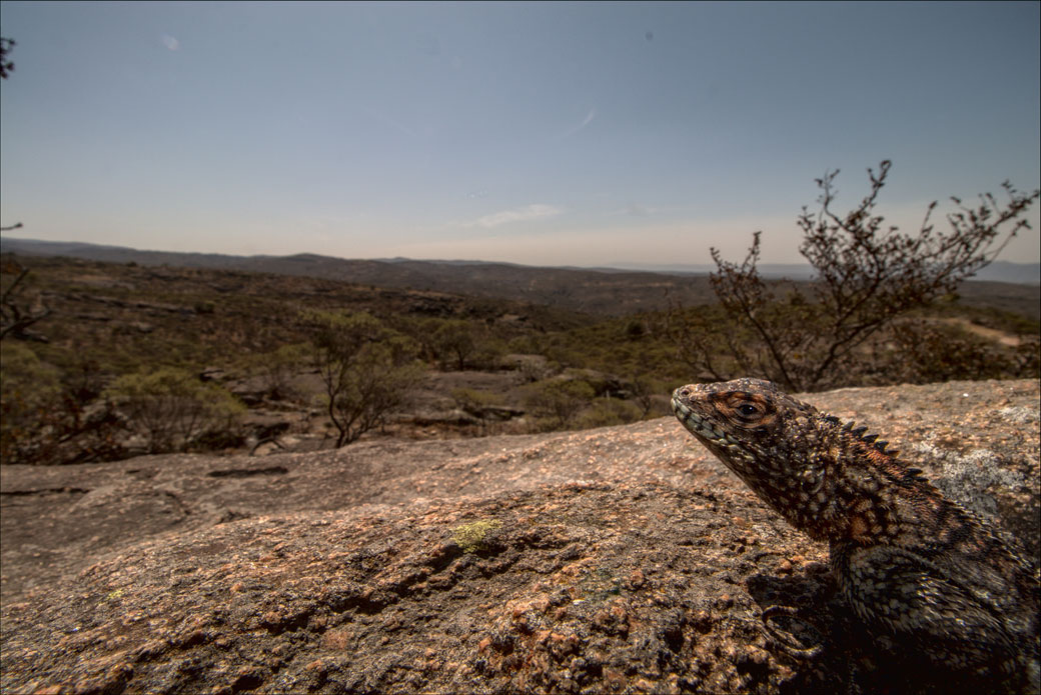
**South American lizard, *Tropidurus spinulosus*, in its natural habitat, the Chaco, which is under threat from global warming.**

**What changes do you think could improve the professional lives of early-career scientists?**

I think that any initiative that helps connecting researchers across the world is always worth considering. To encourage young researchers to interact and expose their ideas could improve their professional life; this opportunity for an interview in Biology Open, for example, is great to boost the visibility of a young researcher. Another pretty common issue I would like to mention is funding, which can become a major problem at times, all the more so for early career researchers. Unfortunately, there is not an easy solution to that either, especially in these times of economic crisis and any new idea/initiative to tackle this issue is more than welcome.

**What's next for you?**

I am currently finishing my PhD thesis and I plan to defend it within this year. Hopefully, I will be tackling the research questions I mentioned above in a post-doc position in the near future.
